# Allele and haplotype frequencies of human platelet and leukocyte antigens in platelet donors

**DOI:** 10.31744/einstein_journal/2019AO4477

**Published:** 2019-02-01

**Authors:** Valeria de Freitas Dutra, Carolina Bonet Bub, Thiago Henrique Costa, Leandro Dinalli Santos, Eduardo Peres Bastos, Maria Giselda Aravechia, José Mauro Kutner

**Affiliations:** 1 Hospital Israelita Albert Einstein, São Paulo, SP, Brazil.

**Keywords:** Human platelet antigen, Platelet refractoriness, Gene frequency, Human leukocyte antigen, Platelet transfusion, Antígenos de plaquetas humanas, Refratariedade plaquetária, Frequência do gene, Antígeno leucocitário humano, Transfusão de plaquetas

## Abstract

**Objective:**

To described the allele and haplotype frequencies of human leukocyte antigen genes at the -A, -B *loci* and human platelet antigen genes for human platelet antigen systems 1 to 9, 11 and 15 in blood.

**Methods:**

We included 867 healthy unrelated volunteer donors who donated platelets between January 2011 and December 2014. Microarray genotyping was performed using a BeadChip microarray. Medium resolution typing of the human leukocyte antigen at *loci* A and B was carried out using sequence-specific oligonucleotide probe hybridization. We used multivariate analysis and our human leukocyte antigen population was compared to data from the United States national bone marrow donor program. Human platelet antigen results were compared to a literature review and data from around the world.

**Results:**

Our human leukocyte antigen haplotype results were more similar to those of hispanics, followed by caucasians. Likewise, our human platelet antigen sample is more similar to those of Argentina, Rio Grande do Sul and Italy.

**Conclusion:**

This was the first article that discusses human platelet antigen and human leukocyte antigen data together. Rare genotypes or antibody associations can make patient management difficult. A blood bank with genotyped donors allows for optimal transfusion and can contribute to better results. Our information can serve as basis for a database of platelet antigen polymorphisms.

## RESUMO

**Objetivo:**

Descrever as frequências alélicas e haplotípicas de genes dos antígenos leucocitários humanos nos *loci* -A,- B e dos antígenos plaquetários humanos para os sistemas HPA-1 a 9, 11 e 15.

**Métodos:**

Foram incluídos 867 doadores voluntários, saudáveis, não relacionados, que doaram plaquetas por aférese entre janeiro de 2011 e dezembro de 2014. A genotipagem foi realizada usando *microarray* BeadChip. A tipificação de resolução intermediária dos antígenos leucocitários humanos *loci* A e B foi realizada por meio de hibridização com sonda para oligonucleotídeos por sequência específica. Utilizamos análises multivariadas e o antígeno leucocitário humano de nossa população foi comparado com a do programa nacional de doadores de medula óssea norte-americano. Já os resultados dos antígenos plaquetários humanos foram comparados à revisão da literatura e a dados de populações de outros países.

**Resultados:**

Os resultados do haplótipo de antígenos leucocitários humanos são mais parecidos com os dos hispânicos, seguidos dos caucasianos. Igualmente, a amostra de antígenos plaquetários humanos foi mais semelhante às da Argentina, do Rio Grande do Sul e da Itália.

**Conclusão:**

Este foi o primeiro artigo a discutir antígenos plaquetários e leucocitários humanos simultaneamente. Genótipos raros ou associações de anticorpos podem dificultar o manejo clínico do paciente. Um banco de sangue com doadores genotipados permite um melhor resultado e transfusão possíveis. Estas informações podem servir de base para um banco de dados sobre polimorfismos de antígenos plaquetários.

## INTRODUCTION

Platelets are anucleated cells, shaped like a discoid lens, which can reach 3-5μm at their greatest diameter.^(^ ^1^ ^)^ Similarly to red and white blood cells, platelets also express many antigens on their surface, which may give rise to immunological issues and hinder the therapeutic effects of a platelet transfusion.^(^ ^2^ ^)^ Human leukocyte antigens (HLA) are glycoproteins (GP) expressed on the surface of nucleated cells, which have a role in tissue rejection. Despite being anucleated, platelets are the major source of HLA class I in the blood. To date, statistics from the Immuno Polymorphism Database (https://www.ebi.ac.uk/ipd/hpa/), a database with allelic records from different populations, show that each platelet can express about 13 thousand molecules of HLA class I: HLA-A, HLA-B and HLA-C, but HLA-C has no clinical importance.^(^ ^3^ ^-^ ^6^ ^)^

Human platelet antigens (HPA) result from single nucleotide polymorphisms (SNPs) in the genes that encode GP expressed on platelet surface membranes. They can form specific antigens that elicit antibodies through exposure to a different platelet.^(^ ^2^ ^)^

When autologous platelets are low and there is active bleeding, platelet transfusion is required for hemostatic control. Also, in a thrombocytopenic patient, platelets are often transfused as a prophylactic treatment before starting any invasive procedure.^(^ ^7^ ^)^ Platelet refractoriness to transfusion is a lack of adequate post-transfusion platelet count increment, which can be calculated by a formula such as, *e.g.* , corrected count increment or percent platelet recovery. It is established after two sequential transfusions using fresh and ABO-identical platelets, from a randomized donor.^(^ ^8^ ^)^ Clinically, refractoriness to platelet transfusion was associated with significantly higher costs, longer lengths of stay, delayed bleeding and poor outcomes following bone marrow transplant for acute myeloid leukemia.^(^ ^8^ ^,^ ^9^ ^)^

About 20% of refractoriness cases are related to immune factors, including HPA and HLA alloimmunization. HLA alloimmunization is more frequent and occurs in about 10 to 20% of cases. The HPA system is less polymorphic than HLA, but the association of anti-HLA and anti-HPA antibodies can pose a difficult problem to blood support. To date, there are 36 HPAs expressed on six different platelet GP: GPIIb, GPIIIa, GPIba, GPIbb, GPIa and CD109.^(^ ^3^ ^,^ ^4^ ^,^ ^6^ ^)^

The probability to find identical HLA donors varies from 10 to 60%, depending on the degree of compatibility applied.^(^ ^10^ ^)^ In Brazil, a mathematical projection model showed that, to find at least five completely compatible donors, 31,940 donors will be necessary to cover 80% of hematological patients. Furthermore, it is not possible to calculate the number of completely compatible donors to cover 100% of patients, because of the great miscegenation.^(^ ^11^ ^)^ Immunization depends on antigen frequency and it can vary among ethnicities. Brazil is an admixed country, where HPA and HLA haplotypes and alleles can influence transfusion results. Our searches did not find any other study about HPA and HLA together and, therefore, our data can improve transfusion practice, offering our patients the best possible platelet match. In addition, this data can be used in genetic and anthropological studies, because it informs on the frequencies of alleles and haplotypes in a Brazilian sample.

## OBJECTIVE

To described the allele and haplotype frequencies of human leukocyte antigen genes at the -A, -B *loci* and human platelet antigen genes for human platelet antigen systems 1 to 9, 11 and 15.

## METHODS

### Subjects

A retrospective study, approved by the Ethics Committee, with data collected from a database. We enrolled 867 healthy volunteer donors who donated platelets between January 2011 and December 2014. Of these, 823 had the HLA, 602 had HPA-1 to 9, 11 and 15; 735 were males; and mean age was 39 years (range: 16 to 66 years). All individuals were unrelated blood donors, accepted for donation after answering a questionnaire and a self-exclusion vote. All had donated at least two platelet aphereses in 1 year.

### Genomic DNA

Peripheral white blood cells from blood donors were isolated by differential centrifugation of whole blood in ethylenediamine tetraacetic acid (EDTA) whole blood. DNA was extracted using a commercial kit (QIAamp blood kit, Qiagen, Valencia, CA), according to the protocol recommended by the manufacturer. The concentration and quality of the DNA obtained were analyzed using a spectrophotometer (NanoDrop™, Thermo Fisher Scientific, San Diego, CA, USA) to ensure the efficacy of extraction and to standardize the DNA amount for PCR.

### Platelet genotyping

Microarray genotyping was performed for HPA systems 1 to 9, 11 and 15, using a BeadChip microarray (Immucor, Warren, NJ, USA). DNA amplification and post-PCR steps were performed following the recommendations of the manufacturer. BeadChip slides were analyzed in a fluorescent microscope, using the BioArray Solutions software (Immucor, Warren, NJ, USA). Intermediate resolution HLA typing of *loci* A and B was carried out using sequence-specific oligonucleotide probe hybridization (SSOPH; One Lambda –Thermo Fisher, Canoga Park, CA, USA).

### Statistical analysis

We used the two-digit HLA nomenclature. Allele and haplotype frequencies were obtained by direct counting. The Arlequin software package 3.5.1.2 was used to calculate allele and haplotype frequencies and gene heterozygosity, and to verify the Hardy-Weinberg equilibrium.^(^ ^12^ ^)^

A multivariate analysis of the data was obtained through hierarchical cluster analysis, comparing our HLA population to the National Marrow Donor Program^®^ (NMDP; www.allelefrequencies.net) population, already categorized as caucasians (n=1,242,890), Africans (n=28,557), Japanese (n=24,582), North American natives (n=187), South or Central American Hispanics (n=146,714), South Asian Indians (n=185,391). We compared our HPA results to data from around the world obtained from the website http://www.ebi.ac.uk/ipd/hpa/freqs_1.html and our literature review. We used: caucasians − Brazil (n=100), African descendants − Brazil (n=150), Amazon Indians − Parakanã tribe (n=70),^(^ ^13^ ^)^ Argentina (n=192), Toba Amerindians (Argentian Indians) (n=27), Ireland (n=250), Benin (n=154), China (n=1,000), Congo (n=125), Cameroon (n=118), French Polynesia (n=81), Italy (n=144), Switzerland (n=500), Vietnam (n=107), Brazil (data from The Immuno Polymorphism Database) (n=400), *Universidade Federal do Rio Grande do Sul* (n=201).^(^ ^14^ ^)^ To illustrate the results we used a dendrogram (HPA-1 to 5). Since the data from comparative populations regarding HPA-6, -9, -11 and -15 were incomplete, these polymorphisms were not included in the multivariate analysis and χ^2^ test, and the Fisher’s exact test was used to compare our sample to others (Statistical Package for Social Science – SPSS, version 22). HPA-7 was monomorphic in all populations from the immune polymorphism website. The significance level was set at p<0.05.

## RESULTS

Table 1 shows the group frequencies for HLA-A (20 alleles) and HLA-B (33 alleles) in the studied donors.

**Table 1:** Allele frequencies of HLA-A and -B in our Brazilian sample

HLA-A	%	HLA-B	%
A*02	26.06	B*35	11.97
A*24	11.42	B*44	10.57
A*03	10.69	B*51	8.14
A*01	9.42	B*15	7.96
A*26	5.22	B*07	6.93
A*11	4.92	B*14	6.38
A*29	4.56	B*08	5.10
A*68	4.56	B*40	4.74
A*23	4.50	B*18	4.37
A*30	4.50	B*38	3.83
A*31	3.65	B*49	3.16
A*33	3.34	B*57	3.10
A*32	2.98	B*50	2.73
A*25	1.34	B*41	2.37
A*74	0.73	B*52	2.37
A*66	0.67	B*39	2.13
A*34	0.49	B*27	2.00
A*36	0.49	B*53	1.88
A*69	0.30	B*45	1.70
A*80	0.18	B*58	1.64
		B*13	1.28
		B*55	1.22
		B*42	1.09
		B*37	1.03
		B*48	0.43
		B*54	0.43
		B*56	0.43
		B*81	0.43
		B*46	0.30
		B*78	0.12
		B*47	0.06
		B*59	0.06
		B*73	0.06

For *locus* A, the allelic groups HLA-A * 80 (0.18%) and -A*02 (26.06%) were the rarest and the most frequent, respectively.

The allelic groups HLA-B*47 (0.06%), -B*59 (0.06%) and -B*73 (0.06%) had low frequencies. Only two allelic groups (HLA-B*35 and -B*44) were found at frequencies above 10%.

When evaluating the genotype distribution for each *locus* , we did not find any deviations from those expected under the Hardy-Weinberg equilibrium (HLA-A, p=0.73; HLA-B, p=0.75).

**Figure 1:**
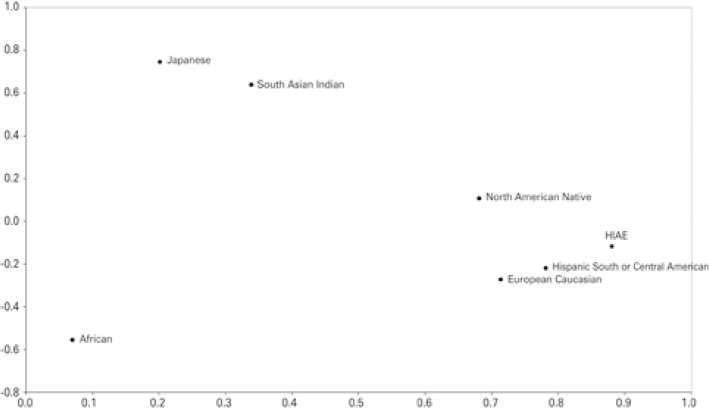
Principal component analysis of HLA haplotypes in our blood donor sample and the National Marrow Donor Program® population

**Figure 2:**
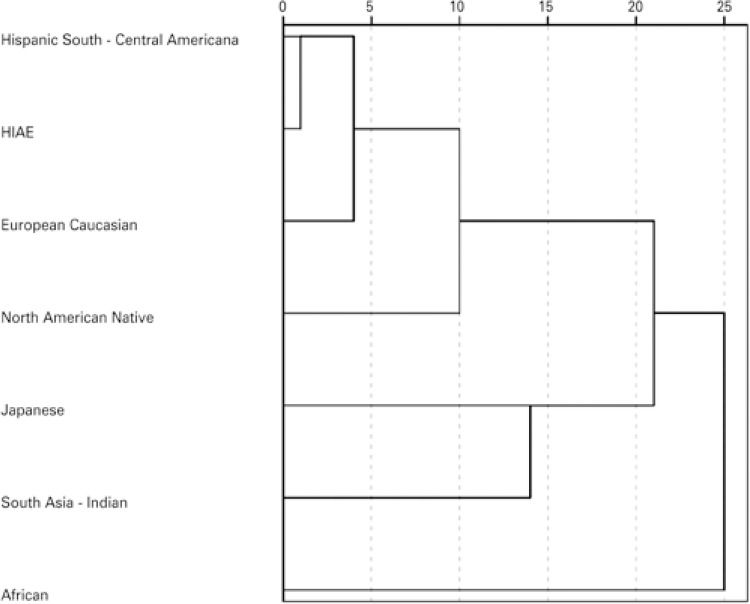
Dendrogram analysis: HLA haplotype comparison between Brazilians and the National Marrow Donor Program® population

We estimated 257 HLA haplotypes. The three more common haplotypes were: A*02 B*44 (4.3%), A*02 B*51 (3.4%) and A*01 B*08 (3.1%). Table 2 shows the 20 most common haplotypes in our study. HLA haplotypes from our blood donors plotted with HLA haplotypes from the NMDP population showed that our sample was closest to hispanics, followed by caucasians. The principal component analysis indicated that the Japanese and African components did not contribute much to our population composition ( Figure 1 ). Genetic proximity was confirmed by the dendrogram: HLA haplotype results, when compared with the NMDP population, were closest to hispanics, followed by caucasians ( Figure 2 ).

**Table 2:** The 20 most frequent HLA haplotypes in our Brazilian sample

Haplotype	%
A*02 B*44	4.32
A*02 B*51	3.37
A*01 B*08	3.07
A*03 B*35	2.79
A*02 B*15	2.48
A*29 B*44	2.39
A*03 B*07	2.15
A*02 B*35	2.12
A*02 B*50	1.80
A*24 B*35	1.72
A*02 B*40	1.71
A*02 B*07	1.59
A*26 B*38	1.56
A*33 B*14	1.51
A*01 B*35	1.37
A*01 B*57	1.34
A*11 B*35	1.30
A*02 B*18	1.19
A*24 B*07	1.13
A*24 B*18	1.04

HIAE: *Hospital Israelita Albert Einstein.*

HIAE: *Hospital Israelita Albert Einstein.*

We typed the HPA system of 602 donors and the allele frequency was, for HPA-1a: 85.7%; 1b: 14.3%; 2a: 88.4%; 2b: 11.6%; 3a: 66.2%; 3b: 33.8%; 4a: 99.9%; 4b: 0.1%; 5a: 88.7%; 5b: 11.3%; 6a: 99.9%; 6b: 0.1%; 7a: 99.9%; 7b: 0.1%; 8a: 100%; 9a: 99.6%; 9b: 0.4%; 11a: 100%; 15a: 100%.

When evaluating the genotype distribution at each *locus* , *loci* HPA-8 and -11 were monomorphic. All other *loci* , except for HPA-1 and -5, were under Hardy-Weinberg equilibrium.

A total of 71 HPA haplotypes were estimated. The most common HPA haplotype was: HPA-1aa; 2aa; 3aa; 4aa; 5aa; 6aa; 7aa; 8aa; 9aa; 11aa; 15ab (9.9% of cases).

**Figure 3:**
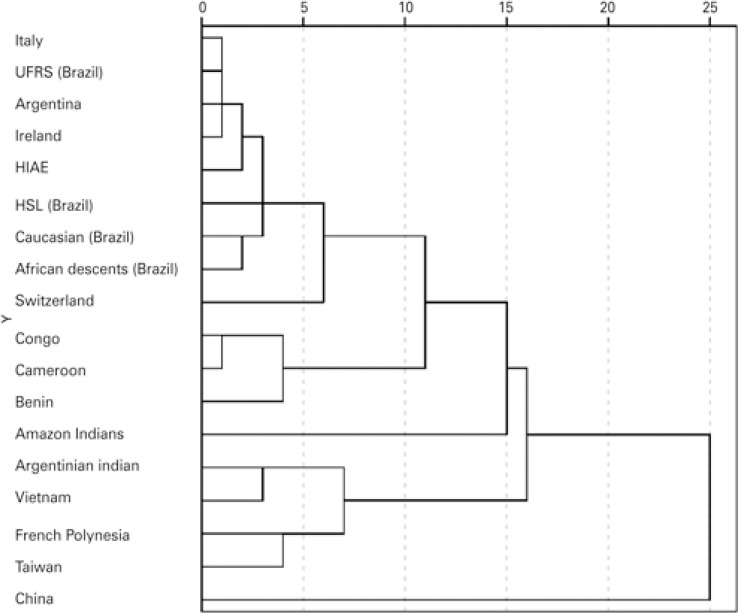
Dendrogram analysis of the HPA allele (HPA-1 to HPA-5) comparison between ours and other populations

UFRS: *Universidade Federal do Rio Grande do Sul* ; HIAE: *Hospital Israelita Albert Einstein* ; HSL: *Hospital Sírio Libanês* .

**Figure 4:**
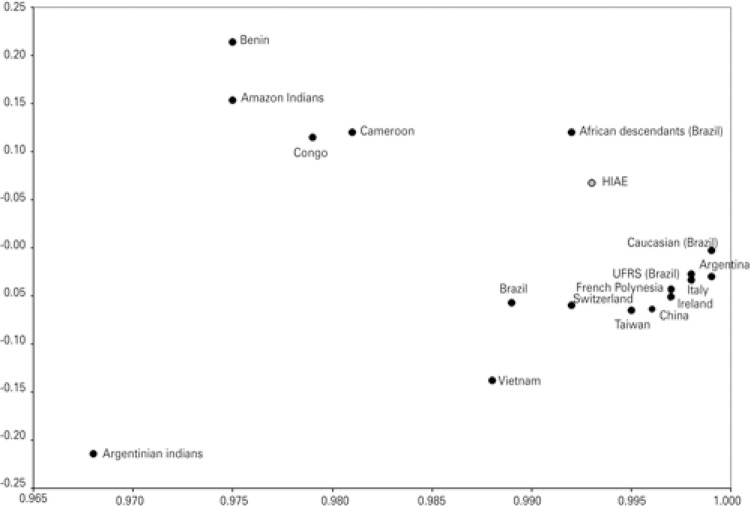
Principal component analysis of HPA alleles

UFRS: *Universidade Federal do Rio Grande do Sul* ; HIAE: *Hospital Israelita Albert Einstein* .

When compared to other populations, our sample is similar to that of Rio Grande do Sul (proximity matrix 1.85), Italy (proximity matrix 2.4), Argentina (proximity matrix 2.967) and Ireland (proximity matrix 3.318). This is shown in figure 3 . When compared to African descendants, our population is not so distant (proximity matrix 4.392), however our sample is distant from Amazon Indians (proximity matrix 18.767). The most distant population were Argentinian Indians (proximity matrix 41.109). The principal component analysis pictured us as a more isolated group ( Figure 4 ). HPA-6 showed statistical differences when compared to the French Polynesia. HPA-15 was different from Argentina, China, Congo, Cameroon and Benin. HPA-9 and -11 showed no differences when compared to different groups (data not showed).

## DISCUSSION

For *locus* A, the allelic groups HLA-A * 80 (0.18%) and -A*02 (26.06%) were the rarest and the most frequent, respectively. HLA-A*02 is a frequent allele and it is spread around the world. It can be as frequent as 47.4% in Argentina, 45.7% in Paraná Mulato or 61.2% in the Chinese population.^(^ ^15^ ^)^ The data also corroborate a previous study in which HLA-A*02 was frequent in Jordanian and five major population groups living in the United States.^(^ ^16^ ^,^ ^17^ ^)^ In the *Registro Nacional de Doadores de Medula Óssea* (REDOME) HLA-A*02 is also the most frequent among different Brazilian ethnicities.^(^ ^18^ ^)^ Rodrigues et al., analyzed the HLA gene frequencies in 366 polytransfused patients, from different regions in Brazil, and found the same result.^(^ ^19^ ^)^

The allelic groups HLA-B*47 (0.06%), -B*59 (0.06%) and -B*73 (0.06%) had low frequencies. Only two allelic groups (HLA-B*35 and -B*44) were found at frequencies above 10%. Such high frequencies resembled those reported for Europe and European-colonized countries.^(^ ^17^ ^,^ ^20^ ^,^ ^21^ ^)^ We found 20 different HLA-A and 33 HLA-B. In the United States, Cao et al., using high resolution DNA typing, found more variation than us. The number of alleles identified in each ethnic group ranged from 25 to 35 for HLA-A and 47 to 63 for HLA-B. There was not an apparent correlation between the number of alleles identified in each population and the size of the population tested.^(^ ^17^ ^)^ A study conducted in the state of Piaui, northeastern region of Brazil, also showed HLA-B*35 and HLA-B*44 at frequencies above 10%, but the most frequent was HLA-B*15.^(^ ^22^ ^)^

A pratical use of this knowledge about HLA is to choose donors using virtual matching. The experience of an Irish blood bank showed that for routine use, the HLAMatchmaker, a computerized algorithm, available free-of-cost online, is effective for selecting units of incompletely matched platelets for alloimmunized and thrombocytopenic patients. The use of this kind of software is increasing in transfusion practice.^(^ ^23^ ^)^

As for the HPA system, an Indonesian study showed that alloimmunization against HPA-1, 2 and 6 is extremely rare; on the other hand HPA-1 is the most important alloantigen in caucasians. The authors did not find donors homozygous for HPA-1b, -2b and -6b.^(^ ^24^ ^)^ We found HPA-8 and 11 in homozygosity, and any of the other HPAs can be a risk for alloimmunization, or else an opportunity to find a rare donor.

In Germany, HPA from Turkish and Caucasian populations was compared and there were no statistical differences between the groups.^(^ ^25^ ^)^ In Pakistan, Bhatti et al., observed a Hardy-Weinberg equilibrium deviation towards alleles HPA-3b and HPA-5b, which was attributed to higher consanguinity rates, which is not common in Brazil.^(^ ^26^ ^)^ Another bias of Hardy-Weinberg expectations can be the subdivision among subpopulations.^(^ ^20^ ^,^ ^26^ ^)^

In 2010, a Brazilian study compared blood bank donors with Amazon Indians. Statistical significance was found for HPA-1, HPA-2, HPA-5 and HPA-15.^(^ ^27^ ^)^ Silvestre et al., found that Japanese descendants from the state of Paraná (southern Brazil) had a HPA-1b allele frequency different from that of the Japanese population, maybe because 40% of them reported to have one parent from Japanese origin and the other from mixed Brazilian origin.^(^ ^28^ ^)^ In the state of Rio Grande do Sul, an analysis of HPA-1 through -5 and HPA-15 showed similarities with the European population.^(^ ^14^ ^)^ Our population is distant from Amazon Indians, but closer to the results of other studies from Brazil, Argentina and Italy. This is in agreement with the history of Brazil and São Paulo. In general, our population’s HLA or HPA is closest to that of Caucasians, which is in agreement with other Brazilian studies. Pimenta et al., found an absence of significant genetic differentiation in a population from São Paulo, classified by phenotype analysis.^(^ ^29^ ^)^ Likewise, in REDOME, it is not possible to segregate ethnicities based on HLA polymorphisms.^(^ ^18^ ^)^ There may be bias in all Brazilian studies, and some populations may be underrepresented in those samples.

Anyway, in a context of admixture, and considering immunological issues, it is important to know the frequency of HPA and HLA alleles and haplotypes to provide the best platelet transfusion to our patients, especially to those who are refractory or alloimmunized.

Today, new light has been shed on the influence of HPA, including the relation between HPA-3 and susceptibility to acute cellular rejection after liver transplantation, and the association between HPA-2 and febrile non-hemolytic transfusion reaction, and this can influence our future decisions.^(^ ^30^ ^,^ ^31^ ^)^

## CONCLUSION

We described the allele and haplotype frequencies of HLA genes at the -A, -B *loci* and HPA genes for HPA-1 to 9, 11 and 15. This is the first study presenting HPA and HLA data together. Clinically, our results can contribute to prepare a database to help find the best donor, even when there is alloimmunization or refractoriness. Rare genotypes or antibody associations can make patient management difficult and having data from genotyped donors can help ensure an adequate post-transfusion platelet count increment.

Therefore, our information can serve as a basis for a database of platelet antigen polymorphisms. By applying this knowledge, since 2014 our blood bank has provided the best platelet match to patients with refractoriness or other history of alloimmunization.
